# Tropical Q Fever

**DOI:** 10.4269/ajtmh.22-0372

**Published:** 2022-07-11

**Authors:** Gilbert J. Kersh

**Affiliations:** Rickettsial Zoonoses Branch, Centers for Disease Control and Prevention, Atlanta, Georgia

Infection with the Gram-negative bacterium *Coxiella burnetii* can result in a variety of clinical presentations that are collectively referred to as Q fever. These can range from a mild, self-limiting febrile illness to more insidious chronic infections such as Q fever endocarditis. *C. burnetii* infections have been found in most of the world, with Europe, Australia, and North America seeming to have the majority of cases. In areas where surveillance is conducted for Q fever, it is a disease that most would consider rare. The United States has conducted national surveillance for Q fever since 2000 and reports an annual average incidence of 0.036 cases per 100,000 persons for 2008 through 2017.^1^ The European Centre for Disease Prevention and Control reported 0.2 Q fever notifications per 100,000 persons for the European Union/European Economic Area in 2019.^2^ Despite the low numbers of reported cases, many areas report fairly high levels of seroprevalence, suggesting that asymptomatic exposures are common.

The baseline circumstance of widespread asymptomatic infections with a minority of infections resulting in detected disease makes the occurrence of exceptional outbreaks notable. Perhaps the most notable of such outbreaks occurred in the Netherlands between 2007 and 2010, where more than 4,000 acute Q fever cases were recorded in a limited geographic area.^3^ During this outbreak, most cases presented with pneumonia. Other, more limited outbreaks have been reported in a variety of locations, including England, Germany, and Bosnia.^4^^–^^6^

The status quo of low baseline prevalence with occasional time-limited outbreaks makes the story of Q fever in French Guiana all the more compelling. French Guiana is a French department on the northeast coast of South America just north of the equator. Estimates of the incidence of Q fever in French Guiana for the late 1990s and early 2000s range from 10 to 40 cases per 100,000 persons per year—much greater than Europe, the United States, and Australia.^7^ There are several unique aspects to the epidemiology of Q fever in French Guiana. In most situations, *C. burnetii* is transmitted by inhalation of the bacteria after shedding by infected livestock. However, in French Guiana, infections do not appear to be linked with exposure to livestock, but capybaras and the three-toed sloth have been implicated as potential reservoirs.^7^ Another feature is that the acute infections tend to be more severe in French Guiana, with pneumonia appearing in 90% of the patients. A unique genotype (MST17) has also been observed in specimens tested from French Guiana.^8^

The latest findings in this emerging story are presented in this issue by Epelboin et al.^9^ They have performed a retrospective case–control study of patients with community-acquired pneumonia (CAP) in the hospital in Cayenne, French Guiana, in the years 2009 through 2012. Q fever cases were compared with patients with either unknown etiologies or etiologies other than *C. burnetii*. This is a follow-up to a similar study reported in 2012 that examined CAP in French Guiana between 2004 and 2007. That study found that 24% of 131 CAP patients evaluated were diagnosed with Q fever.^10^ This is a much higher percentage than that reported from other regions, which typically find 0% to 3% of CAP patients with a Q fever diagnosis. Even at the peak of the outbreaks in the Netherlands (2007–2009) only 12% to 22% of CAP was a result of Q fever. The high percentage of Q fever CAP in French Guiana in 2004 through 2007 was notable, but the question remained as to whether this was an unusual outbreak or whether high endemic levels of Q fever were a consistent leading cause of CAP in this region. The current study found that over a 4-year period (2009–2012), 38.5% of 275 patients with CAP were diagnosed with Q fever, with no clear trend of increasing or decreasing numbers of Q fever cases.^9^ The results suggest that French Guiana had high endemic levels of Q fever that commonly presented as pneumonia for an extended period. Unfortunately, the results presented reflect the situation ≥ 10 years ago, and information on the current situation in French Guiana is unavailable.

The study also highlights some of the difficulties with diagnosis, as the authors have difficulty applying a straightforward case definition for Q fever.^9^ Sixteen of the 106 patients with Q fever CAP were identified based on a single positive serology, which could have been from a previous infection. The authors rely on the opinion of the treating physician to determine whether these patients with only a single positive serology had Q fever. The inclusion of patients with single serology results and the fact that testing for other etiologies was only done at the discretion of the attending physician could inflate the numbers somewhat, as opinions could be biased by previous studies describing Q fever as a major etiology for CAP in this hospital. However, even with some overestimation, the high number of cases at a single hospital raises clinical suspicion of Q fever among CAP patients to a high enough level to influence treatment protocols.

Although the current study did not address incidence directly, it once again suggested that Q fever incidence in French Guiana is much higher than that seen in Europe and the United States. The drivers of high incidence will undoubtedly be multifactorial and may include the unique ecology of *C. burnetii* in the region, but one likely contributor to the high percentage of Q fever CAP is the high virulence of the MST17 genotype.^8^ The situation that is commonly observed of widespread clinically mild *C. burnetii* infections with only a subset getting diagnosed could be shifted toward more severe cases on average in French Guiana as a result of the MST17 genotype, allowing cases to be identified more easily. The relative stability of the Q fever situation in other countries could rely on the prevalence of relatively low-virulence strains in livestock populations. Monitoring and understanding *C. burnetii* genotypes in local animal reservoirs could therefore provide an indicator of impending changes in Q fever disease incidence and severity.

Q fever is rarely reported from other countries in Latin America, and is not commonly recognized in Africa. Certainly, the attention of the Institut Pasteur and the French National Reference Center for Q Fever has been instrumental in identifying the situation in French Guiana. Their results suggest that the incidence of Q fever may be much higher in tropical areas than is currently recognized. This is supported by a recent estimate of Q fever annual incidence in northern Tanzania of 80 cases per 100,000 persons.^11^ The preponderance of Q fever cases in Europe, Australia, and North America could merely reflect greater availability of resources in these regions. Improvements in the availability of diagnostic testing, epidemiological expertise, and health-care provider education are all key components for a better understanding of Q fever in tropical areas.
